# Human adipose-derived mesenchymal stem cells accelerate decellularized neobladder regeneration

**DOI:** 10.1093/rb/rbz049

**Published:** 2019-12-22

**Authors:** Victoria Moreno-Manzano, Maravillas Mellado-López, Maria Jose Morera-Esteve, Ana Alastrue-Agudo, Viviana Bisbal-Velasco, Jerónimo Forteza-Vila, Ángel Serrano-Aroca, César David Vera-Donoso

**Affiliations:** 1 Neuronal and Tissue Regeneration Lab, Centro de Investigación Príncipe Felipe, Valencia, Spain; 2 Facultad de Veterinaria y Ciencias Experimentales, Universidad Católica de Valencia San Vicente Mártir, c/Guillem de Castro 94, Valencia 46001, Spain; 3 Surgery Department, Veterinary Faculty, Universidad CEU, Valencia, Spain; 4 Molecular Pathology and Translational Research in Oncology, Unidad Mixta Universidad Católica de Valencia y Centro de Investigación Príncipe Felipe, Spain; 5 Department of Urology, La Fe University and Polytechnic Hospital and Health Research Institute, Hospital La Fe, Valencia 46026, Spain

**Keywords:** mesenchymal stem cells, bladder regeneration, decellularized matrices, neobladder

## Abstract

Decellularized natural bladder matrices (neobladders) represent an exciting means to regenerate the bladder following bladder cancer-associated cystectomy. In this study, we compare the evolution of decellularized matrices with recellularized matrices by seeding it with human adipose-derived mesenchymal stem cells (ADSC) after implantation following partial cystectomy in rats. We discovered significant anatomical differences since 10 days after neobladder implantation with the ADSC-containing matrices promoting a significant recovery of mature p63- and cytokeratin 7-positive urothelium. We also discovered significantly induced expression of the vimentin mesoderm marker in the submucosal layer in ADSC-seeded matrices. Interestingly, we found a higher expression of smooth muscle actin in transversal and longitudinal smooth muscle layers with ADSC-seeded matrices. Furthermore, ADSC also showed increased vascularization and nerve innervation of the neobladder as determined by the distribution of CD31 and S100β reactivity, respectively. We believe that ADSC and their paracrine-acting pro-regenerative secretome within decellularized matrices represent an efficient bladder substitution strategy; however, we require a fuller understanding of the mechanisms involved before clinical studies can begin.

## Introduction

Acquired and congenital bladder abnormalities currently lack effective treatment strategies for organ substitution, and radical cystectomy remains as the current standard treatment for localized muscle-invasive bladder cancer [1]. This process involves the complete removal of bladder, prostate and pelvic lymph nodes in males, and the bladder, ovaries, uterus and a portion of the vagina in females, followed by the permanent diversion of urine through the abdomen using a separated piece of bowel as an incontinent external diversion (ileal conduit) or using an internal diversion (‘neobladder’) [2, 3]. However, numerous complications occur with approaches using gastrointestinal segments (including mucus production, recurrent urinary tract infections, electrolyte imbalances, etc.) due to the incompatibility of this type of tissue with urine [1]. Overall, the replacement of urinary bladder tissue with functional equivalents remains a challenging problem in reconstructive urology.

As an alternative strategy, tissue engineering approaches can avoid these problems and achieve successful results in bladder reconstruction, including the application of decellularized matrices for bladder substitution [2]. Studies have proven natural decellularized matrices to be a valuable tissue engineering option for whole tissue and bladder regeneration, providing enhanced functional outcomes when compared to other tested synthetic biomaterials, partly due to immune recognition and lower risk for host-versus-graft reactions [3, 4]. These natural matrices are rich in collagen; however, this slowly degrades after implantation and is replaced by proteins from the extracellular matrix, thereby supporting regeneration without immune rejection [5]. In this regard, it has been shown that the presence of collagen enriched hydrogels obtained from digested decellularized matrices, like for instance the ones obtained after bladder decellularization, influence on the inflammatory response by a polarization of the recruited macrophages, switching from a pro-inflammatory phenotype (M1) into an anti-inflammatory one (M2) which will be in favor of the cell transplantation tolerance [6]. Decellularized matrices preserve matrix composition and retain chemical and biological cues and a micro-compartmentalized structure similar to the native cellular tissue, thereby rendering these types of scaffolds a hospitable environment for cell repopulation [7]. The most widely employed decellularized matrices in bladder replacement typically derive from either the bladder itself or small intestinal submucosa; both strategies have proven successful results in several animal models (reviewed in [8]). The application of said matrices first requires a reliable decellularization process [9] followed by the intricate steps involved in cell repopulation, cell organization and cell fate specification while retaining functional capabilities.

The bladder performs repeated and coordinated contractions thanks to the specific characteristics of the smooth muscle fibers and their sophisticated organization in perpendicular layers, displaying connections with blood vessels and innervations through peripheral nerves through the complex matrix without compromising the storage capacity of the bladder or protection of the upper tract [10, 11]. The bladder transitional epithelia or urothelium is a specialized epithelium comprising a stratified tissue of basal, intermediate and superficial cell layers which work together to maintain a protective barrier against water, ions and pathogens with specific resistance to the acidic nature of urine [12]. The connective tissue provides the fibrillary scaffold for blood and peripheral nerve end distribution and the storage of nutrients [13]. Therefore, all specialized tissues have to be taken into account for complete or partial bladder replacement.

Stem cell therapies have shown to be a promising approach to enhance the regeneration of tissue-engineered bladder tissue [14]. Adipose tissue-derived mesenchymal stem cells (ADSC) represent an easy to obtain and abundant stem cell source with low immunogenicity and huge potential autologous applications. Studies have already demonstrated the potential of ADSC in improving urethral tract function in animal models [15] and in a model of neobladder reconstruction by recellularized matrix bladder [16] and porous synthetic scaffolds [17, 18]. Furthermore, ADSC therapy represents a safe and feasible means to treat conditions related to the urothelial tract in humans [19]. However, the underlying mechanisms of action remain controversial, an increasing body of evidence suggests the paracrine effect of stem cells, rather than cell differentiation, as the main factor for the regeneration of functional tissues [20]. Additional analysis of bladder tissue regeneration mediated by ADSC would help to improve therapeutic results and herein, we establish the capacity of ADSC as part of a recellularized bladder matrix to contribute to bladder tissue regeneration after partial cystectomy by bladder substitution.

## Materials and methods

### Bladder matrix decellularization

Bladders were dissected from adult female Sprague-Dawley rats weighing 250–300 g. Immediately after dissection, bladders were washed three times in phosphate-buffered saline (PBS) containing 1% of streptomycin/penicillin (S/P; Invitrogen) and then transferred to tris buffer (10 mM, pH 8) solution containing 1% sodium dodecyl sulfate (SDS; Sigma Aldrich) and incubated overnight at 4°C, shaking at 400 rpm. The bladders were then immersed in 0.5% Triton X-100 (Invitrogen) in Tris solution (10 mM, pH 8) for 4 h and then transferred to ammonia hydroxide solution (0.05%) in Tris buffer (10 mM, pH 8) for 2 h, shaking at 400 rpm. Bladders were repeatedly washed in sterile PBS containing S/P before being transferred to 70% ethanol for ten minutes and then stored in PBS-S/P at 4°C until transplantation or the re-cellularization process.

### Human ADSC isolation, culture and recellularization of decellularized bladder matrices

Human adipose tissue was obtained from surplus fat tissue during knee prosthesis surgery of four patients under sterile conditions. The human samples were anonymized, and the experimental procedure previously evaluated and accepted by the Regional Ethics Committee for Clinical Research with Medicines and Health Products following the Code of Practice 2014/01. As exclusion criteria, no samples were collected from patients with a history of cancer or infectious diseases at the time of the surgery (viral or bacterial). All human patients voluntarily signed an informed consent document for the use of the adipose samples. ADSC were isolated, expanded and characterized as previously described [21, 22]. ADSC were maintained and expanded in growth medium containing 10% fetal bovine serum (FBS) in DMEM (Dulbecco's modified Eagle Medium) medium containing 2 mM L-glutamine, 1% L-glucose and P/S. ADSC at passages three to four were used for the recellularization of decellularized bladders. A suspension of 500 μl containing approximately 1 × 10^6^ ADSC (stained with PKH26 Red Fluorescent Cell linker for general cell membrane labeling following manufacturer instructions (Mini26-1kt 028K0431, Sigma)) was employed for bladder recellularization. Four hundred microliters of the ADSC suspension was injected into the bladder matrix submucosa using a 30G-Hamilton syringe (84852, Postnova, USA), with the remaining 100 μl of ADSC suspension deposited into the lumen of the bladder matrix. The recellularized matrix bladders were individually cultured under sterile conditions, wholly covered with growth medium for 5 days *in vitro* (DIV). Bladders were manually rotated every 2 h during the first DIV and twice a day during the following days. Three bladders were employed for *in vitro* assays, including cell distribution and phenotypic analysis at 5 DIV. Phalloidin (P1951 de Sigma) staining for *in vitro* bladder fluorescence analysis was performed after overnight fixation of bladders with PFA (paraformaldehyde) 4%, and incubation for 1 h at room temperature at a 1/100 dilution. Whole bladders were mounted using Fluor Save Reagent (Calbiochem, USA) and the fluorescence signal of both Phalloidin and the red fluorescent cell linker was visualized by Confocal Microscopy (Leica, Germany).

### Neobladder implantation

Experimental animals were bred at the Animal Experimentation Unit of the Research Institute Príncipe Felipe (Valencia, Spain), where the experimental protocol was previously approved by the Animal Care Committee in accordance with the National Guide to the Care and Use of Experimental Animals (Real Decreto 1201/2005). Adult Sprague Dawley female rats were subdivided into two groups (i) for decellularized bladder matrix implantation and (ii) for 5 DIV ADSC recellularized bladder matrix implantation (*n* = 3/group). The key surgical steps are illustrated in Supplementary Fig. S1. Under isoflurane anesthesia and opioid analgesia (morphine, 2.5 mg/kg body weight, Braun, Germany), the native bladder (employed for comparative histological analysis) was removed before bladder implantation (nearly complete cystectomy). The neobladders were attached to the remnant urethra with a non-absorbable double needle-suture (5-0 Prolene, Ethicon-Johnson & Johnson, Florida, USA) and were covered with peritoneal adipose tissue. The abdomen was closed in two layers. Animals were treated with antibiotic (Enrofloxacin, 5 mg/kg body weight/day for 7 days; Ecuphar, Germany) and buprenorphine (0.1 mg/kg body weight/12 h for 4 days; Indivior UK Limited, UK) and meloxicam (0.2 mg/kg body weight/day for 3 days; Boehringer Ingelheim, Germany) for 7 days after implantation. Cyclosporine (10 mg/kg of body weight/day; Novartis, Spain) was subcutaneously administered until the animals were sacrificed at 10 days after bladder implantation. Before sacrifice, 10 or 20 days after implantation, the bladders were ecographically visualized (Supplementary Fig. S2; General Electric, VIVID 7 Pro, USA).

### Histological analysis and immunoassays

Bladder matrices, both decellularized or recellularized and from *in vitro* assays (5 DIV) or from *in vivo* experimentation were fixed with 4% PFA for 4 h, then washed in PBS, and embedded in paraffin. Deparaffinized and hydrated sagittal slices of 4 μm tissue sections were stained with hematoxylin–eosin (Coverstainer, Dako) or Masson Trichrome (MT) (Artisanlinkl pro, Dako). For immunohistochemistry (IHQ), the paraffin-embedded sections were first deparaffinized, processed for antigen retrieval by incubation in citric acid-based un-masking solution (Vector Laboratories), permeabilized with a PBS solution containing 0.1% Triton X-100, and blocked with 5% goat serum in PBS for 1 h. The following primary antibodies were diluted in blocking solution and incubated for 60 min at room temperature at 1:100 dilutions: monoclonal mouse anti-P63 (IR662), anti-Cytokeratin 7 (IR619), anti-smooth muscle actin (SMA) (IR611), anti-Desmin (IR606), anti-Vimentin (IR630), anti-S100β (IR504), anti-CD31 (IR610) and anti-Ki67 (IR626) from Dako or anti-human mitochondria (MAB1273) from Chemicon. After being rinsed three times with PBS, cells were incubated with an HRP (horseradish persoxide)-conjugated goat anti-mouse IgG-HRP secondary antibody, for 40 min at room temperature, and the DAB (3,3'-Diaminobenzidine) substrate kit (Envision Dako) performed in an automatic autostainer link 48 (Dako). Both, anatomical stainings and IHQ were scanned in a Panoramic 250 Flash II scanner (3DHISTECH Ltd.; HUNGARY) and images acquired with the Panoramic viewer software. Quantification of the images was performed with Image J, expressed in px^2^ or the percentage of positive cells and normalized to the total analyzed area.

### Statistical analysis

Results were reported as the mean ± standard error of the mean as indicated for each set of data. For the comparisons between groups, statistical analysis of the results was performed by the one-way ANOVA, with appropriate corrections such as Tukey’s *post hoc* test was used. Statistical analyses were performed using GraphPad software. Differences were considered significant at *P values < 0.05; **P <* *0.01; ***P <* *0.001; ****P <* *0.0001.

## Results

### *In vitro* recellularization of decellularized bladder matrix with human ADSC

**Figure 1:**
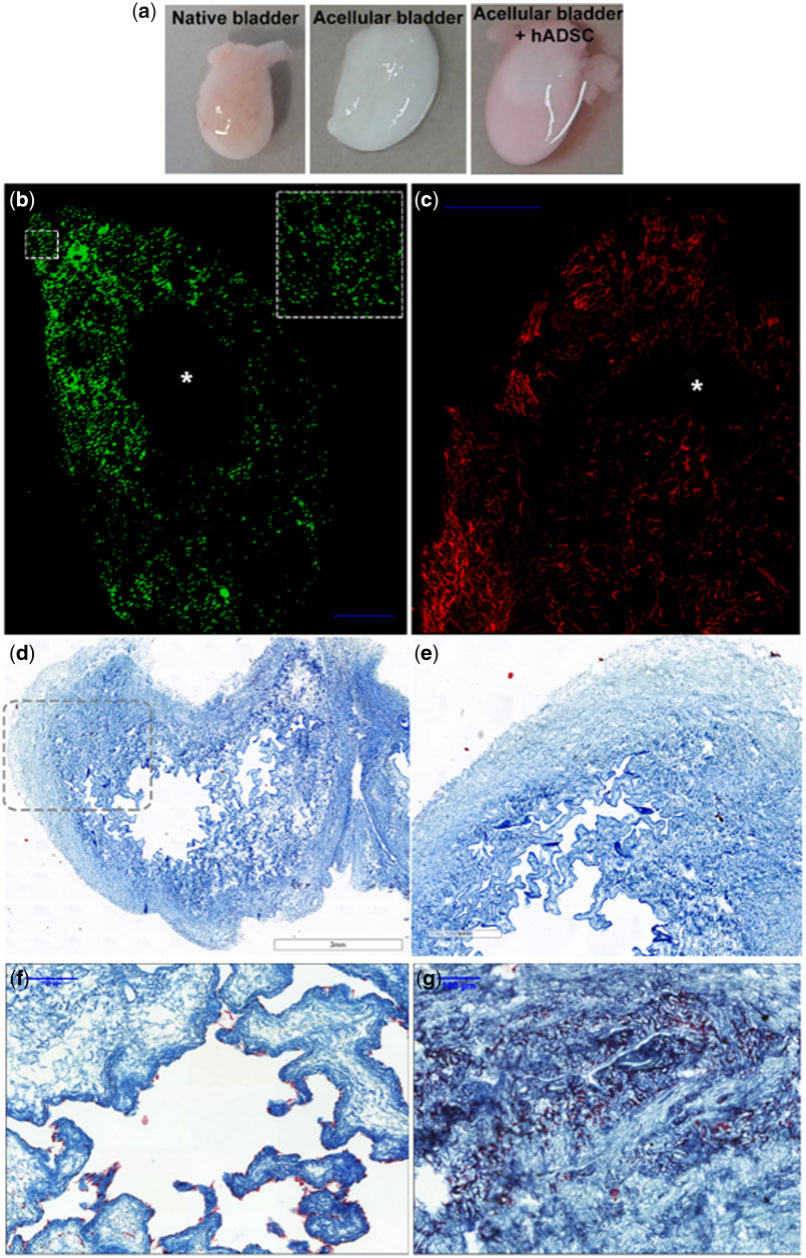
*In vitro* recellularization of bladder matrices with human ADSC. (**a**) Macroscopic view of a dissected native rat bladder (left panel), decellularized bladder after decellularization process (central panel) and decellularized bladder recellularized with labeled human ADSC (right panel); (**b**) confocal images of the *in vitro* recellularized matrix bladder with ADSC labeled with cell tracker [green (pseudo-color), left panel] and (**c**) phalloidin (red, right panel) and cultured for 5 DIV demonstrate a homogeneous cell distribution along the collagen-rich matrix. The inset square in (b) depicts a magnification of the indicated area. The internal lumen of the bladder is empty (*); (**d** and **e**) Masson’s trichrome staining of decellularized bladder matrix, (e) enlarged image of dotted line marked area in (d); (**f** and **g**) trichrome staining of neobladders after 5 DIV demonstrates the contrast in the collagen-rich matrix stained in blue-green and the ADSC in purple-red lined within the urothelial layers (f) and growing in clusters in the mucosa and submucosa areas (e). Scale bar, in blue: 100 μm.

We decellularized native adult rat bladders via three consecutive washing steps in Tris buffer containing first SDS (1%), then Triton X-100 (0.5%) and ammonium hydroxide solution (0.05%) and then recellularized rat bladders with approximately 1 million human ADSC. We distributed 90% of these cells by direct injection into the matrix wall with a Hamilton syringe (30G) to cover the entire bladder, and then deposited the final 10% into the bladder lumen (Fig. 1a). We next cultured recellularized bladders in the presence of 10% FBS in standard cell conditions for 5 DIV. Figure 1b depicts the uniform distribution of ADSC, labeled immediately before re-cellularization with the PKH26 fluorescent cell linker membrane marker (Fig. 1b, green) through the whole bladder mucosa and submucosa surrounding the hollow lumen (Fig. 1b, *). We also observed extensive F-actin fibers labeled with phalloidin (Fig. 1c, red) after 5 DIV.

MT staining of sagittal sections in Fig. 1d and e shows the decellularized bladder matrix structure, absent of cellular content. The *in vitro* recellularized bladders demonstrated a concatenated alignment of the deposited ADSC in a single layer in the lumen with a flat cell shape attached to the external wall of the former urothelial stratum (Fig. 1f). We found ADSC clusters on the internal bladder wall, as evidenced by robust purple staining within the collagen enriched bladder matrix (Fig. 1g).

### *In vitro* cell fate analysis of ADSC in recellularized bladders matrices

**Figure 2:**
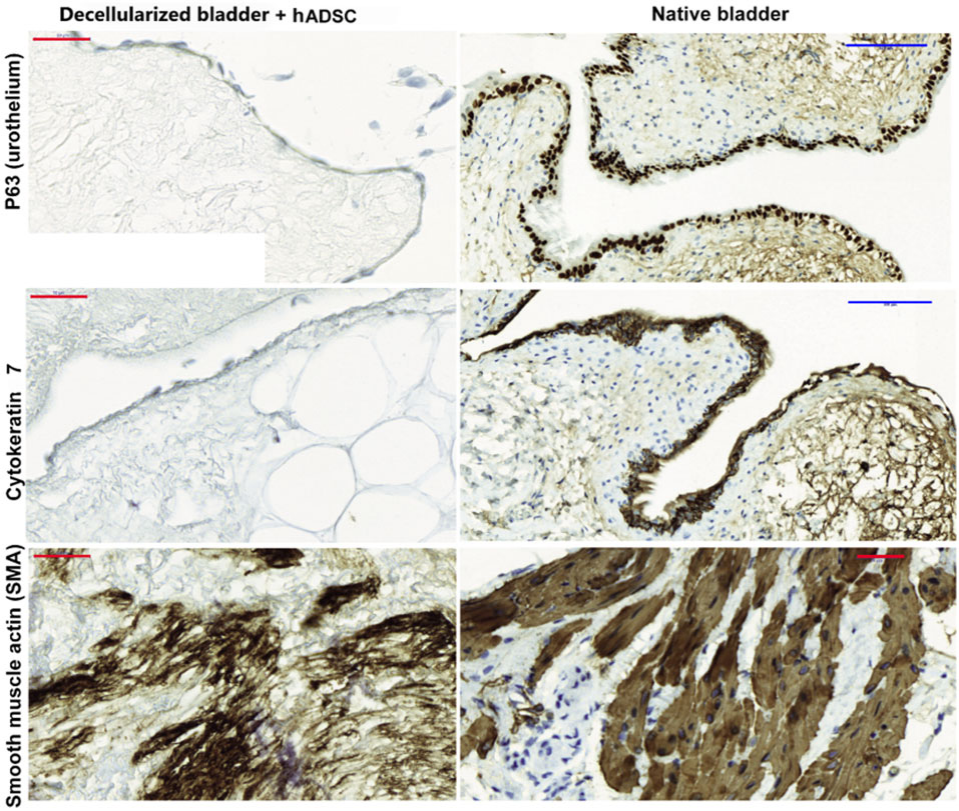
ADSC differentiate into smooth muscle fibers in the decellularized matrix bladder *in vitro*. The bladder matrices recellularized with ADSC after 5 DIV were fixed in 4% PFA and embedded in paraffin. Four micrometer sagittal sections were used to assess the distribution of the p63 and cytokeratin 7 epithelial markers (upper panels). SMA expression was used for smooth muscle-specific lineage detection (lower panel) expression in the native bladder is shown in the right panels to compare with those from the *in vitro* regenerated bladder (left panels). Scale bars: blue: 100 μm, red: 50 μm.

While ADSC lining the internal wall exhibited an epithelial-like shape, they displayed low protein expression level of p63, a urothelial marker [23], and cytokeratin 7, an epithelial marker [24] when compared to the native bladder (Fig. 2, left upper panels). However, we observed robust expression of SMA, a smooth muscle marker [25] in those ADSC found forming clusters in the submucosa/mucosa matrix, indicating that signals from the matrix specify a specific lineage differentiation route (Fig. 2, left and lower panels). The expression levels of p63, cytokeratin 7 and SMA in the native bladder (Fig. 2, right panels) evidenced the immature stage of the cells integrated into the bladder matrix. The recellularized bladder generally lacked p63 and cytokeratin 7; however, the presence of high SMA levels indicates a preference for smooth muscle cell specification.

### Recellularization of bladder matrices with ADSC promotes neobladder tissue regeneration after *in vivo* implantation

We implanted 5 DIV recellularized or decellularized bladder matrices immediately after partial cystectomy in female adult rats (Supplementary Fig. S2 depicts a sequential image reconstruction of the surgical procedure). We sacrificed transplanted rats 10 or 20 days after implantation; during these days, we made a daily inspection of rats for potential urinary infections. We manually emptied transplanted bladders twice daily using the credé maneuver. All rats transplanted with the recellularized bladder matrices retained urine from Day 7 after implantation; however, all control rats transplanted with decellularized matrices bladders showed wet abdomens until sacrifice time due to the loss of urine.

**Figure 3:**
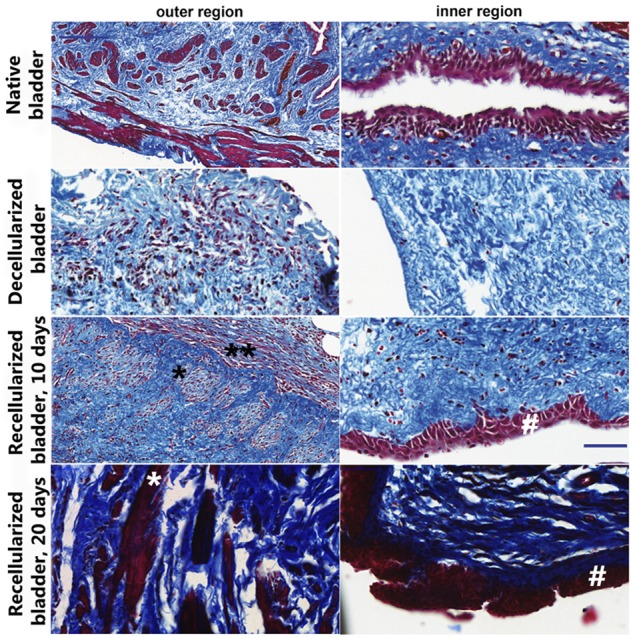
Cell invasion, differentiation and tissue organization of implanted matrix bladders. Ten or 20 days after implantation of the *in vitro* recellularized (lower panels) or decellularized bladder matrices (central panels) cellularization was compared to the native bladder (upper panels) after MT staining. The implanted decellularized bladder matrices displayed the poorest cellularization with prominent cell invasion only at the mucosal region (central panels). The implanted recellularized bladder matrices were explored at 10 or 20 days after implantation and displayed stratified urothelial layer (#) and a clustered cell population within the former smooth muscle layers (longitudinal organization, *; transversal organization, **). Scale bar: blue: 100 μm.

We next performed Masson’s trichrome staining to analyze dissected bladders. Both, decellularized and recellularized bladder matrices were invaded by endogenous cells (stained in purple) within the matrices (stained in blue) since 10 days after implantation, although each displayed a different cell distribution and tissue organization (Fig. 3). As shown in Fig. 3, the implanted neobladders failed to completely recover the physiological tissue organization at earlier stages, 10 days after implantation; however, the recellularized matrix analyzed 20 days after implantation showed a closer tissue maturation stage of the native bladder (Fig. 3). The recellularized bladder matrices consistently displayed the formation of new detrusor and inner muscle layer structure with circular bundles of clustered cells (*) and longitudinal layers of fusiform like-smooth muscle fibers (**; Fig. 3, lower-left panels) more compacted and with more mature morphological appearance in the recellularized bladder analyzed 20 days after implantation. Cells infiltrating into the decellularized bladder matrices displayed a random distribution without a defined pattern (Fig. 3, central panels). Importantly, only the recellularized bladder matrices displayed the formation of the transitional stratified urothelium since 10 days after implantation (#; Fig. 3, lower-right panels).

### ADSC accelerate specific tissue regeneration in transplanted neobladders

**Figure 4:**
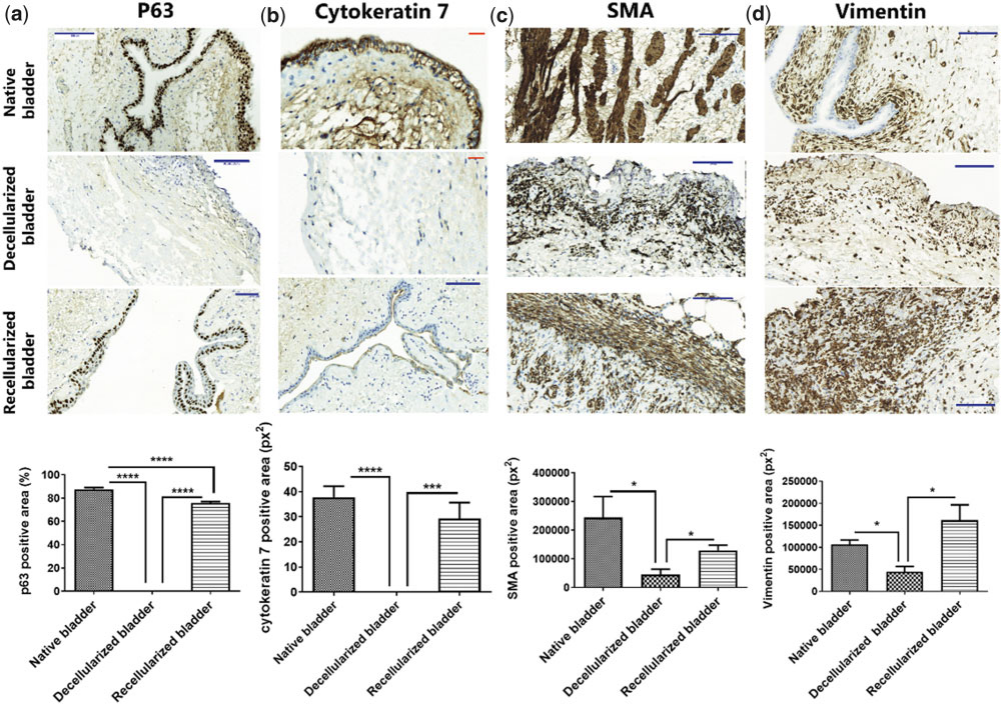
Recellularization of decellularized bladder matrices with ADSC accelerates endogenous bladder regeneration *in vivo*. *Upper panels*: (**a**) Representative images of p63 immunostaining (urothelial marker) and (**b**) cytokeratin 7 (epithelial cell marker) demonstrates a stratified transitional epithelium in the recellularized bladder matrices compatible with the native bladder. Ten days after implantation, the decellularized bladder matrices failed to demonstrate positive staining in the former urothelial layer; (**c**) Representative images of SMA specific staining in longitudinal and transversal orientations at the submucosa in the recellularized bladder matrices demonstrate a comparable muscle layer organization to that of native tissue. Ten days after implantation, the decellularized bladder matrices exhibit an SMA-positive population of cells lacking a defined orientation; (**d**) Representative images for vimentin immunostaining evidence massive mesoderm-derived cell infiltration in the recellularized bladder matrices, mostly located at the external perimeter of the recellularized neobladder. Ten days after implantation, the decellularized bladder matrices exhibit few vimentin-positive cells. Scale bars: red: 100 μm, blue: 50 μm; *lower panels*: graphical quantification. Data are expressed as mean ± SEM. *P < 0.05; ***P < 0.001; ****P < 0.0001.

To assess the nature and maturation stage of the regenerated cell layers found in the implanted neobladders, we performed immunohistochemical staining for p63 (urothelial cell marker) (Fig. 4a), cytokeratin 7 (epithelial cell marker) (Fig. 4b), SMA (smooth muscle fiber marker (Fig. 4c)) and vimentin (mesoderm marker) (Fig. 4d) 10 days after bladders implantation in order to investigate the cell fate specification at the earlier regenerative stages.

We failed to detect P63 staining in the decellularized bladder matrices, in accordance with the absence of any cell stratification at the former urothelial layer (Fig. 4a); however, we discovered a significant induction of P63 expression in the recellularized bladder matrix (Fig. 4a). Although we observed a stratified cell layer compatible with transitional urothelial tissue structure in the recellularized bladder matrices, the lack of a significant number of cells positive for P63 indicated an immature phenotype for this newly developed urothelium (Fig. 4a). We obtained comparable results after cytokeratin 7 staining (Fig. 4b).

SMA staining indicated the existence of infiltrating cells at the former detrusor and inner muscle layers in both decellularized and recellularized bladder matrices, indicating robust differentiation-inducing signaling from the extracellular matrix (Fig. 4c). However, we discovered a significantly higher density of SMA-positive cells in the recellularized bladder matrices and a well-organized fiber orientation when compared to the decellularized bladder matrices (Fig. 4c).

We employed vimentin, an intermediate filament present in the mesoderm-derived cells [26], to quantify the enrichment in connective tissue cells. We found that recellularized bladder matrices displayed a significantly higher number of vimentin-positive cells, concentrated at the mucosa and submucosa areas when compared to native bladders, where abundant vimentin-positive cells were found immediately beneath the epithelial layer (Fig. 4d). The widespread invasion of vimentin-positive cells was induced by the presence of the ADSC, as the decellularized bladder matrices exhibited a significantly lower level of mesoderm cell infiltration (Fig. 4d).

**Figure 5:**
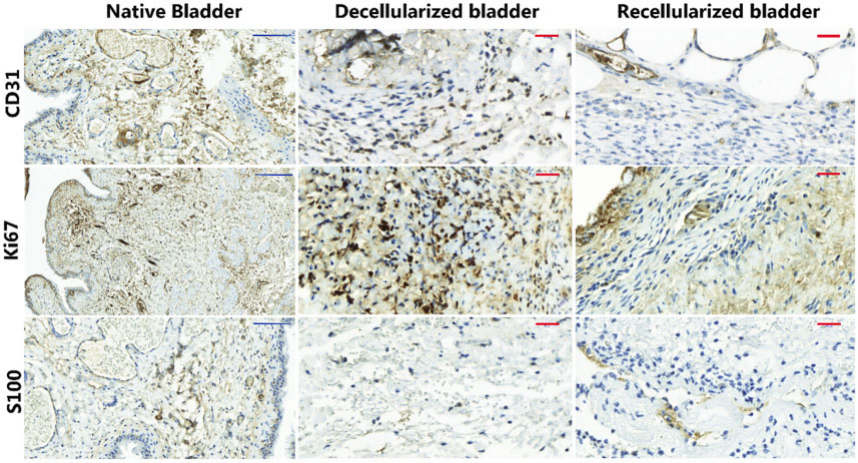
ADSC increase vascularization and nerve innervation in the implanted neobladders. Representative images of immunohistochemically analysis of the CD31 endothelial marker for blood vessels (upper panels), the Ki67 cell proliferation marker (central panels) and the S100β peripheral nerve end marker (lower panels) in decellularized (left panels) and recellularized (right panels) bladder matrices 10 days after implantation. Scale bars: blue: 50 μm; red: 100 μm.

We evaluated whether ADSC in the recellularized matrices increased the vascularization process by using the CD31 endothelial marker [27]. The recellularized bladder matrices displayed, by qualitative evaluation, more blood vessels with a mature architecture (Fig. 5a, upper panels); however, the quantification of the CD31 positive cells did not reveal a significant difference between all the experimental groups, decellularized vs recellularized matrices (data not shown), as the decellularized bladder also exhibited a high number of CD31-positive cells within the connective tissue without displaying a blood vessel-like structure. Staining for Ki67, a mitotic marker [28], indicated the active proliferation of cells within the decellularized bladder matrices in comparison with the recellularized ones (Fig. 5, central panels) in concordance with the observed inflammatory active process. Interestingly, staining for the S100β peripheral nerve end marker [29] was almost absent in the decellularized bladder matrices with only a disperse distribution; in contrast, the recellularized bladder matrices displayed abundant positive staining organized in clusters indicative of an active re-innervation process (Fig. 5, lower panels).

## Discussion

Since the end of the 19th century, the search for an ideal urinary diversion strategy that complements cystectomy has been arduous and, unfortunately, unsuccessful [30]. Currently, autologous segments of gastrointestinal tissue are generally employed for bladder replacement to restore bladder storage and voiding capacity [31]. The most used techniques are ileal conduit urinary diversion, orthotopic bladder substitution or continent cutaneous diversion. Several characteristics, including its simple mobilization, extensive mesentery and general abundance make the ileum the most frequently utilized segment in bladder augmentation; however, after one century, the ileum remains the gold standard solution. Although the removal of the bladder is relatively an uncomplicated technique, the involvement of the intestinal tract for the transport of urine generates a complication rate greater than 50%. Additionally, involved patients are usually elderly, which makes post-operative management complex.

Human ADSC integrated into the decellularized bladder matrix provide support and induce endogenous urinary bladder tissue regeneration after *in vivo* implantation. We tested the capacity of human ADSC into a murine model of bladder cystectomy for further translational applications. We observed significantly improved tissue regeneration for the implanted ADSC-containing neobladders, immediately after almost complete cystectomy, when compared to implantation of the decellularized matrix bladder.

Decellularized matrices fabrication process has been perfected to better maintain the 3D architecture after matrix decellularization, and preserve the unique niche protein composition, mechanical properties and bioactive molecules to serve as perfect substitutes scaffold for tissue repair (reviewed in [32]). Chemical, physical and enzymatic procedures would be used individually or in combination. Here, we decellularized the bladder matrices in three consecutive steps using low concentration of ionic detergents (SDS 1%) for gently cell removal, and nonionic detergents (Triton X-100 0, 5%) for lipid interactions removal leaving protein–protein interactions within the extracellular matrix [33]. Rat bladder matrices decellularization process was easily addressed by sequential immersion for a day on hypotonic containing detergent solutions (Fig. 1a); however, bladders from large animals would need the combination of strategies, including anterograde and retrograde perfusion of chemical decellularization agents through the vascular network during several days to ensure complete decellularization (reviewed in [34]).

Decellularized matrices have been shown to induce cell differentiation of their former tissue [35]. Bladder matrix strongly induced mesoderm-like specific phenotypes of the seeded ADSC after 5 DIV (Fig. 2); ADSC differentiated into smooth muscle fibers when were located at the former detrusor layers (see Supplementary Fig. S3 for native bladder anatomical overview). However, the ADSC did not induce the formation of the urothelium *in vitro*. Nevertheless, a simple concatenated layer of flat cells was located at the outermost layer of the forms transitional epithelium, exhibiting very low expression levels of cytokeratin 7, that displayed the primitive epithelial cell fate of seeded and migrating ADSC. Previous studies reported the need to culture ADSC with conditioning medium from primary urothelial cultures to induce a urothelial phenotype [36]; here, we provide evidence that the matrix alone can induce a primitive epithelial phenotype.

The regeneration of the urothelium is the most challenging task in urinary tract cell therapy. Nevertheless, the combination of the proper matrix and the transplantation of undifferentiated ADSC could provide significant benefits to patients. The implantation of neobladders previously seeded with ADSC for 5 DIV led to a significant recovery in the stratified urothelium since 10 days post-implantation (Figs 3 and 4a and b). However, inspection by immunostaining using human mitochondria antibody of the implanted human ADSC suggests a low survival rate since few cells were detected 10 days after implantation (data not shown), therefore, suggesting the activation of endogenous responses by ADSC-derived paracrine-acting factors, such a urothelial chemoattractant signaling to the niche to induce proliferation and migration of the epithelial precursors. Wolffian duct-derived epithelial cells at the urethra can support urothelial bladder regeneration [37], and as the cystectomy was incomplete in this case, the remnant host urethra could contribute to the repopulation of the neobladder following stimulation after implantation of the ADSC-containing matrix.

Smooth muscle fibers formed in a longitudinal and transversal organization at the former detrusor location were found in the implanted recellularized bladder and not the bladder matrix (Fig. 4c). However, 10 days after implantation the fibers were either abundant or compacted in comparison with 20 days after sacrifice time after bladder implantation (Fig. 3). The bladder matrix induced a fast-smooth muscle identity within the first week after implantation in the rat; however, organization into longitudinal forms with circular boundaries and compact smooth muscle fibers need the presence of ADSC indicating an influence of paracrine-acting secreted factors. Indeed, one study has reported that only the ADSC secretome drives the rescue of urinary function more efficiently than when compared to the presence of the cells [38].

Of note, the implanted animals failed to control the vesical reflex until the end of the study after almost complete cystectomy. Macroscopic observation after neobladder dissection demonstrated a significant contracture (shortening of a muscle) with calcifications in the decellularized neobladders (data not shown), an issue identified as the major limitation during the regeneration process [39]; however, we failed to see this in the recellularized bladders. It has been hypothesized that the lack of angiogenesis is the primary cause of contracture [39]. Also, the histological hallmarks found in the recellularized bladders showing preferential cluster organization of blood vessels and nerve end (Fig. 5) would be compatible with an efficient a functional tissue reorganization.

All in all, decellularized bladder matrices *in vitro* recellularized with ADSC are suitable for bladder substitution. ADSC in the decellularized bladder matrices reconstitute the urothelium and smooth muscle layers’ organization with limited inflammatory invasion for a better functional vesical reflex regeneration.

## Funding

This research was funded by AEI RTI2018-095872-B-C21/ERDF and the Fundación de Urología Pedro Cifuentes. 

## Supplementary Material

rbz049_Supplementary_Data
