# Supplementary material for: Human adipose-derived mesenchymal stem cells accelerate decellularized neobladder regeneration
Source: Regen Biomater. 2019 Dec 22;7(2):161–9. doi: 10.1093/rb/rbz049 (PMC7147364; doi:10.1093/rb/rbz049)
Supplement: rbz049_Supplementary_Data [file rbz049_supplementary_data.zip › rbz049-Suppl_Data/Figure S1.docx]

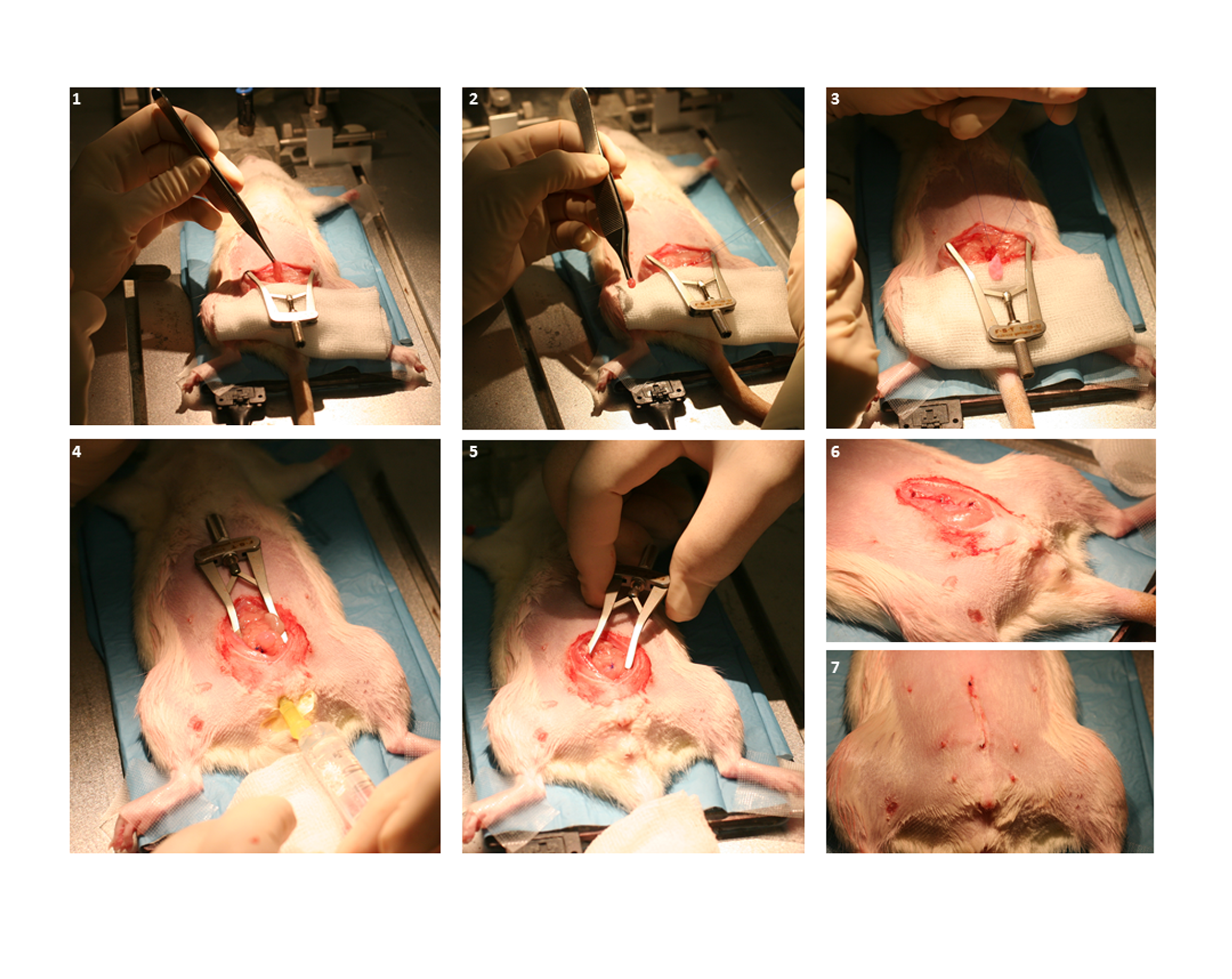


**Figure S1. Surgical procedure for neobladder implantation**. 1) Abdominal incision and bladder localization; 2) Before bladder removal and complete cystectomy (left hand shows the removed bladder), the urethra was suture avoiding occlusion of the lumen (right hand); 3) The neobladder was then sutured to the urethra from both ends with a double needle suture following a Conell suture pattern; 4) the neobladder hermeticity was checked by introducing saline solution through the urethra; 5) The neobladder was enveloped with adipose tissue from the peritoneum; 6) the muscles were saw in planes; 7) the skin was sutured with internal stitches.
