# Supplementary material for: Human adipose-derived mesenchymal stem cells accelerate decellularized neobladder regeneration
Source: Regen Biomater. 2019 Dec 22;7(2):161–9. doi: 10.1093/rb/rbz049 (PMC7147364; doi:10.1093/rb/rbz049)
Supplement: rbz049_Supplementary_Data [file rbz049_supplementary_data.zip › rbz049-Suppl_Data/Figure S2.docx]

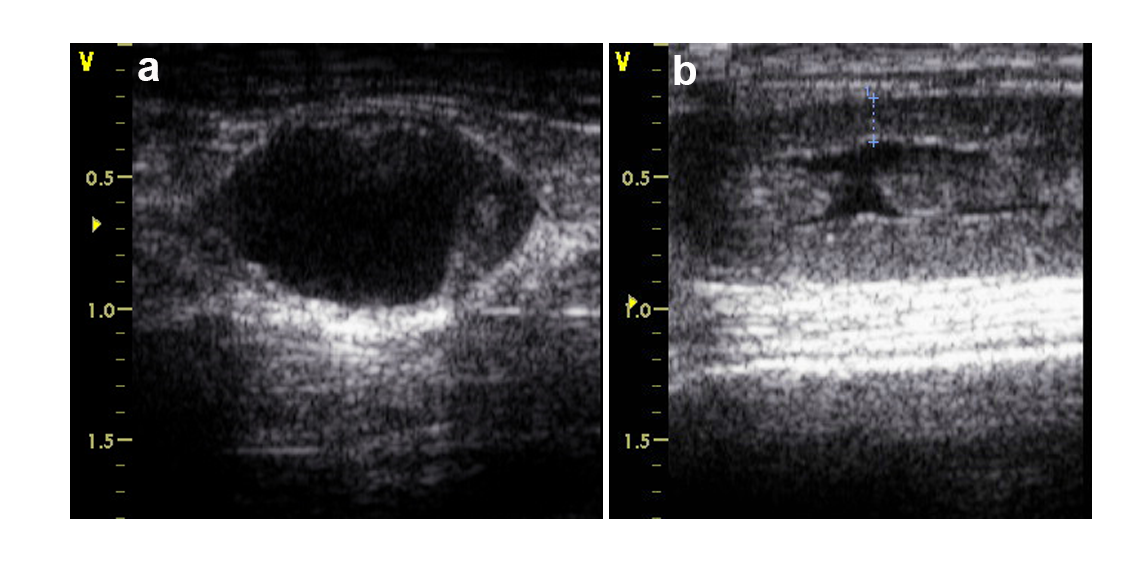


**Figure S2. Ecographic analysis of the implanted neobladder.** 10 days after neobladder implantation, the recellularized bladder in a sagittal section showed an expanded internal cavity, full of urine, with a standard thinness wall in the crosssection.
