# Supplementary material for: Human adipose-derived mesenchymal stem cells accelerate decellularized neobladder regeneration
Source: Regen Biomater. 2019 Dec 22;7(2):161–9. doi: 10.1093/rb/rbz049 (PMC7147364; doi:10.1093/rb/rbz049)
Supplement: rbz049_Supplementary_Data [file rbz049_supplementary_data.zip › rbz049-Suppl_Data/Figure S3.docx]

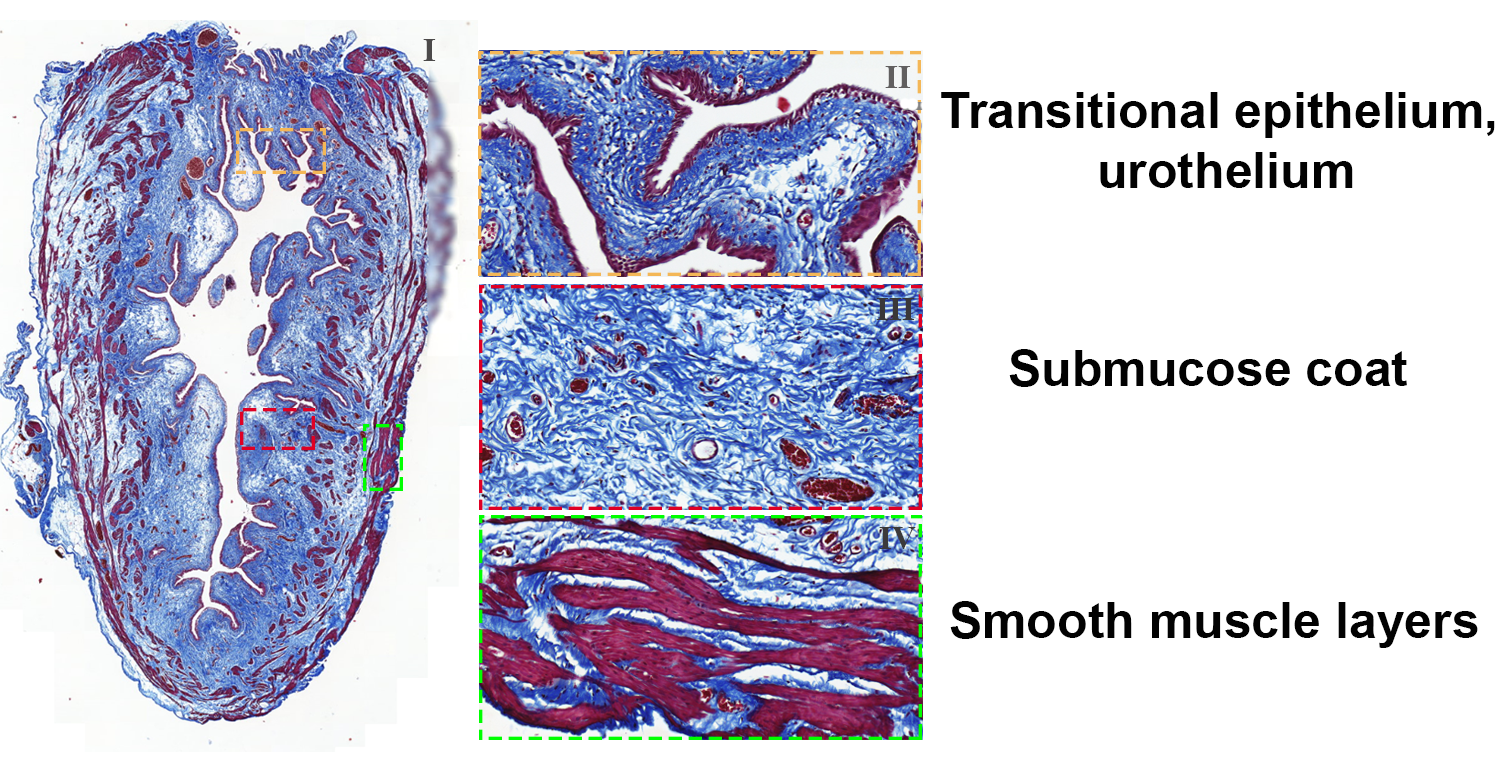


**Figure S3. Urinary bladder histological organization in physiological conditions.** I–IV. Representative images from a longitudinal bladder section from an adult male rat after Trichromic Masson staining. The adventitious layer (the outermost), the urothelium (II) and the muscle bundles (IV) are stained in dark red and the extracellular matrix of the submucosa is stained in blue (III). It is of note that the location of II, III and IV layers are shown in image I by means of a coloured frame with dashed line; V: The illustration on the right shows the bladder organization in layers. Starting from the lumen, bladder is composed by a transitional epithelium or urothelium formed by 4–5 layers of specialized cells supported by the basal lamina and followed by the submucosa coat, loose connective tissue containing fibroblasts, blood vessels and extracellular matrix, enriched in collagen I and III. Bellow, consecutive layers with perpendicular orientations of smooth muscle form first an inner muscle layer and later, the detrusor with smooth muscle fibers organized in circular and longitudinal layers. The adventitious layer of adipose tissue completes the structural organization. *Adapted from Serrano A et al, 2018.*
